# A Systematic Review of Missouri Lymphedema Symptom Assessment Tools in Original Research and Review Articles

**DOI:** 10.1093/geroni/igaa057.649

**Published:** 2020-12-16

**Authors:** Christiana Oyewusi, Jane Armer

**Affiliations:** University of Missouri-Columbia, Columbia, Missouri, United States

## Abstract

Despite advances in cancer treatment, many survivors face a significant challenge of cancer-related lymphedema, together with aging. Aging results in structural changes in the lymphatic system. Beginning in 1998, tools have been developed at the University of Missouri to assess symptoms of lymphedema in cancer patients. The objective of this review was to synthesize evidence regarding use of Missouri lymphedema symptom assessment tools in original research and review articles. A search of six electronic databases was conducted for articles published within 1998 and 2018 on review and use of the tools which are Lymphedema and Breast Cancer Questionnaire (LBCQ), Melanoma and Lymphedema Questionnaire (MELQ), and Gynecologic Cancer and Lymphedema Questionnaire (GCLQ). In all, 210 articles were retrieved, and 32 full-text articles meeting the inclusion criteria were reviewed. The studies reported a cumulative number of 5,872 study participants. Most manuscripts (70.97%) reported data on breast cancer lymphedema, 19.35% on gynecological cancer lymphedema, 3.23% on breast cancer and melanoma lymphedema, and 6.45% on melanoma. The use of LBCQ was reportedly more than the use of GCLQ and MELQ. Tool reliability ranged from r = 0.785 - 0.82 for LBCQ and 0.95 for internal reliability for GCLQ. The tools have been used in many countries including the United States of America. The importance of using valid and reliable quantitative measures in lymphedema symptom assessment across diverse populations and sites cannot be overstated. The tools have been modified and used over the past 20 years in various settings and across languages and cultures.

